# Supplementary material for: Type 2 diabetes worsens the outcome of ischemia/reperfusion in female STEMI patients and female db/db mice with HFpEF cardiometabolic phenotype
Source: Cardiovasc Diabetol. 2025 Jun 7;24:243. doi: 10.1186/s12933-025-02771-z (PMC12144705; doi:10.1186/s12933-025-02771-z)
Supplement: Supplementary file 1 — Supplementary Material 1. [file 12933_2025_2771_MOESM1_ESM.docx]

|  | **Control**  (n=40) | **Diabetic**  (n=40) | P value |
| --- | --- | --- | --- |
| ***3 days post hospital admission*** | | | |
| Erythrocytes (x 10^9^/L) | 4.20±0.07 | 4.24±0.08 |  |
| Haemoglobin (g/L) | 128.9±2.44 | 125.5±1.89 |  |
| Haematocrit (g/L) | 0.38±0.006 | 0.37±0.005 |  |
| Thrombocytes (x10^9^/L) | 253.2±14.8 | 247.8±12.4 |  |
| Leukocytes (x 10^9^/L) | 8.79±0.52 | ***10.01±0.49** | 0.0079 |
| MCV(fL) | 88.55±1.91 | 88.58±0.81 |  |
| Glucose (mmol/L) | 6.04±0.18 | ***11.49±0.68** | 0.0001 |
| Urea (mmol/L) | 6.29±0.40 | 7.63±0.59 |  |
| Creatinine (mmol/L) | 74.03±2.39 | ***93.79±7.47** | 0.018 |
| Potassium (mmol/L) | 4.16±0.08 | 4.36±0.08 |  |
| Na (mmol/L) | 138.5±0.71 | 138.7±0.69 |  |
| Ca (mmol/L) | 2.26±0.02 | 2.32±0.035 |  |
| Mg (mmol/L) | 0.84±0.01 | 0.81±0.02 |  |
| P (mmol/L) | 1.26±0.025 | ***1.12±0.06** | 0.0103 |
| AST (U/L) | 86.84±8.42 | ***63.31±6.42** | 0.0292 |
| ALT (U/L) | 42.36±3.54 | 38.50±3.01 |  |

**Supplementary Table 1. Patient plasma profile 3 days post-hospitalization**

Data are presented as mean ± SEM. Normality of data distribution was examined using Shapiro–Wilk’s normality test. Comparison between two groups was performed by Student’s t-test (Gaussian data distribution) or Mann-Whitney U test when data was non-normally distributed.

| **Culprit vessel**  (number of patients) | **Control**  (n=40) | **Diabetic**  (n=40) |
| --- | --- | --- |
| LM | 1 | 0 |
| LAD | 14 | 16 |
| Prox LAD | 7 | 9 |
| Mid LAD | 6 | 8 |
| Dist LAD | 0 | 0 |
| CX | 3 | 5 |
| Prox CX | 0 | 3 |
| Dist CX | 3 | 1 |
| OM | 1 | 2 |
| RCA | 22 | 17 |
| Prox RCA | 6 | 10 |
| Mid RCA | 14 | 5 |
| Dist RCA | 2 | 2 |

**Supplementary Table 2**. Number of culprit vessels affected by occlusions

LAD – left anterior descending, CX - circumflex, OM – obtuse marginal, RCA – right coronary artery, Prox-proximal, Mid-medial, Dist-distal

**Supplementary Figure 1.** Mouse plasma profile lean vs db/db 20-week female mice. (n=6 lean, n=6 dbdb). Data are presented as mean ± SEM. Normality of data distribution was examined using Shapiro–Wilk’s normality test. Comparison between two groups was performed by Student’s t-test (Gaussian data distribution), unequal variance t-test, or Mann-Whitney U test when data was non-normally distributed, *p<0.05.
